# Phospholipase Cζ rescues failed oocyte activation in a prototype of male factor infertility

**DOI:** 10.1016/j.fertnstert.2012.08.035

**Published:** 2013-01

**Authors:** Michail Nomikos, Yuansong Yu, Khalil Elgmati, Maria Theodoridou, Karen Campbell, Vyronia Vassilakopoulou, Christos Zikos, Evangelia Livaniou, Nazar Amso, George Nounesis, Karl Swann, F. Anthony Lai

**Affiliations:** a Cardiff University School of Medicine, Heath Park, Cardiff, United Kingdom; b National Center for Scientific Research “Demokritos,” Aghia Paraskevi, Greece; c IVF Wales, University Hospital of Wales, Heath Park, Cardiff, United Kingdom

**Keywords:** Fertilization, male infertility, oocyte activation, phospholipase C, PLC-zeta

## Abstract

**Objective:**

To determine the effect of infertility-linked sperm phospholipase Cζ (PLCζ) mutations on their ability to trigger oocyte Ca^2+^ oscillations and development, and also to evaluate the potential therapeutic utility of wild-type, recombinant PLCζ protein for rescuing failed oocyte activation and embryo development.

**Design:**

Test of a novel therapeutic approach to male factor infertility.

**Setting:**

University medical school research laboratory.

**Patient(s):**

Donated unfertilized human oocytes from follicle reduction.

**Intervention(s):**

Microinjection of oocytes with recombinant human PLCζ protein or PLCζ cRNA and a Ca^2+^-sensitive fluorescent dye.

**Main Outcome Measure(s):**

Measurement of the efficacy of mutant and wild-type PLCζ-mediated enzyme activity, oocyte Ca^2+^ oscillations, activation, and early embryo development.

**Result(s):**

In contrast to the wild-type protein, mutant forms of human sperm PLCζ display aberrant enzyme activity and a total failure to activate unfertilized oocytes. Subsequent microinjection of recombinant human PLCζ protein reliably triggers the characteristic pattern of cytoplasmic Ca^2+^ oscillations at fertilization, which are required for normal oocyte activation and successful embryo development to the blastocyst stage.

**Conclusion(s):**

Dysfunctional sperm PLCζ cannot trigger oocyte activation and results in male factor infertility, so a potential therapeutic approach is oocyte microinjection of active, wild-type PLCζ protein. We have demonstrated that recombinant human PLCζ can phenotypically rescue failed activation in oocytes that express dysfunctional PLCζ, and that this intervention culminates in efficient blastocyst formation.

**Discuss:** You can discuss this article with its authors and with other ASRM members at **http://fertstertforum.com/nomikosm-plc-zeta-oocyte-activation-male-infertility/**

Oocyte (or egg) activation, the earliest step of mammalian embryonic development after fertilization, is triggered by a characteristic series of large cytoplasmic Ca^2+^ transients known as Ca^2+^ oscillations (1, 2). This striking Ca^2+^ signaling phenomenon is both necessary and sufficient for the completion of all the events of egg activation such as cortical granule exocytosis, which acts to prevent polyspermy, the resumption and completion of meiosis, and pronuclei formation (3). Over the last decade, there has been growing evidence indicating that the physiologic agent responsible for generating Ca^2+^ oscillations and the subsequent egg activation is a testis-specific isoform of phospholipase C, PLCζ (4–7). These studies culminate in the proposal that PLCζ is delivered by the fertilizing sperm into the ooplasm, whereupon it hydrolyzes the membrane phospholipid substrate, phosphatidylinositol 4,5 bisphosphate (PIP_2_), to trigger cytoplasmic Ca^2+^ oscillations via the inositol 1,4,5-trisphosphate (InsP_3_) intracellular Ca^2+^ signaling pathway (3–5). The smallest known mammalian PLC isozyme of ∼70 kd, PLCζ consists of four EF hands, the catalytic X and Y domains, and a C2 domain (4, 5). Each of the individual PLCζ domains appears to have an essential role in conferring the distinct biochemical characteristics and the unique mode of regulation of this gamete-specific PLC isozyme (8–14).

The fundamental role of PLCζ in mammalian fertilization has been further highlighted by recent clinical studies that have directly linked abnormal PLCζ protein expression profiles with documented cases of male infertility (15–18). Sperm from patients that displayed either reduced PLCζ protein abundance or that expressed mutated forms of PLCζ were correlated specifically with failed fertilization after intracytoplasmic sperm injection (ICSI) treatment, which was due empirically to the inability of such sperm to initiate the vital Ca^2+^ oscillations required for egg activation (15–18). The observation of aberrant sperm PLCζ protein expression in infertile males suggests that the wild-type PLCζ protein could be used as a potential therapy to overcome such cases of infertility. However, it is not known whether the wild-type human PLCζ protein is able to physiologically activate eggs in the presence of mutant PLCζ and if this would successfully lead to normal embryo development.

We now show that purified recombinant human PLCζ protein is capable of hydrolyzing PIP_2_ with a similar Ca^2+^ dependence to mouse PLCζ, and that it can also induce cytoplasmic Ca^2+^ oscillations after microinjection into both mouse and human eggs, leading to successful egg activation and early embryo development. We also demonstrate the deleterious effect of male-infertility-linked PLCζ mutations on both Ca^2+^ oscillations and PIP_2_ hydrolysis activity. Notably, mouse eggs expressing the mutant human PLCζ were unable to activate normally and failed to commence embryo development. However, this infertile phenotype could be effectively rescued by microinjection of the wild-type human PLCζ protein, leading to Ca^2+^ oscillations and successful early embryogenesis up to the blastocyst stage. Our findings demonstrate the potential utility of PLCζ protein in in vitro fertilization (IVF) treatment, thus providing a novel therapeutic agent that may help to overcome those cases of egg activation failure caused by deficient or defective forms of PLCζ in human sperm.

## Materials and methods

### Expression Plasmid Construction and cRNA Synthesis

A pCR3 plasmid construct encoding human PLCζ-luciferase (19) was subjected to site-directed mutagenesis (QuikChange II; Stratagene) to generate the PLCζ^H233L^ and PLCζ^H398P^ mutants. Wild-type human PLCζ (GenBank #AF532185) and the H233L and H398P mutants were amplified by polymerase chain reaction (PCR) from the corresponding pCR3 plasmid by use of Phusion polymerase (Finnzymes) to incorporate a 5′ SalI site and a 3′ NotI site and were cloned into a modified pET expression vector (pETMM60). The primers used for amplification of wild-type and mutant PLCζ were: 5′-CCTAGTCGACATGGAAATGAGATGGTTTTTGTC-3′ (forward) and 5′-CTAAGCGGCCGCTCATCTGACGTACCAAACATAAAC-3′ (reverse).

Similarly, mouse PLCζ (GenBank #AF435950) was amplified by PCR from a pCR3-mouse PLCζ-luciferase construct (8) in the same manner and cloned into pETMM60. The mouse PLCζ primers used were: 5′-CTCAGTCGACATGGAAAGCCAACTTCATGA-3′ (forward) and 5′-ATCAGCGGCCGCTCACTCTCTGAAGTACCAAAC-3′ (reverse). Rat PLCδ1 (GenBank #M20637) was amplified by PCR from a pGEX-5X2-PLCδ1 construct (8) in the same manner and cloned into pETMM60. The rat PLCδ1 primers used were: 5′-CTCAGTCGACATGGACTCGGGTAGGGACTTCC-3′ (forward) and 5′-ATCAGCGGCCGCTCAGTCCTGGATGGAGATCTT-3′ (reverse). After linearization of the various luciferase-tagged PLC plasmid constructs, complementary RNA (cRNA) encoding the respective PLC was synthesized (13, 14) by use of the mMessage Machine T7 kit (Ambion) and a poly(A) tailing kit (Ambion), as per the manufacturer's instructions.

### Protein Expression and Purification

For the NusA-PLC fusion protein expression studies, *Escherichia coli* (Rosetta [DE3]; Novagen) transformed with the appropriate pETMM60 plasmid was cultured at 37°C until *A*_600_ reached 0.6, and NusA-fusion protein expression was induced for 18 hours at 16°C with 0.1 mM isopropyl-β-D-thiogalactopyranoside (IPTG; Promega). Cells were harvested (6,000 × *g* for 10 minutes), resuspended in phosphate-buffered saline (PBS) containing a protease inhibitor mixture (EDTA-free; Roche), and sonicated 4 × 15 seconds on ice. Soluble NusA-fusion proteins were then purified by affinity chromatography on Ni-NTA resin after standard procedures (Qiagen) and elution with 275 mM imidazole. Eluted proteins were dialyzed overnight (10,000 MWCO; Pierce) at 4°C against 4 L of PBS, and concentrated with centrifugal concentrators (Sartorius; 10,000 MWCO).

### Assay of PLC Activity

The PIP_2_ hydrolytic enzyme activity of recombinant PLC proteins was assayed as previously described elsewhere (8, 11, 13). The final concentration of PIP_2_ in the reaction mixture was 220 μM, containing 0.05 μCi of [^3^H]PIP_2_. The hydrolysis assay conditions were optimized for linearity of enzyme kinetic activity, requiring a 10-minute incubation of 20 pmol of PLCζ protein sample at 25°C. In assays to determine dependence on PIP_2_ concentration, 0.05 μCi of [^3^H]PIP_2_ was mixed with cold PIP_2_ to give an admixture of the appropriate final PIP_2_ concentration. In assays examining PLC Ca^2+^ sensitivity, Ca^2+^ buffers were prepared by EGTA/CaCl_2_ admixture, as previously described elsewhere (8, 13).

### Preparation of Gametes and Analysis of Embryos

Experiments were carried out with mouse eggs in HEPES-buffered potassium simplex optimized medium (H-KSOM) as previously described elsewhere (8, 12, 14). Eggs obtained from superovulated mice were microinjected 14.5 to 15.5 hours after human chorionic gonadotropin (hCG) administration (14, 19). All procedures were in accordance with the UK Home Office Animals Procedures Act and were approved by the Cardiff University Animals Ethics Committee.

For the egg activation and embryo development studies, recombinant human PLCζ protein-injected mouse eggs were kept in KSOM containing 5 μg/mL cytochalasin B for 6 hours. After pronuclei formation was observed, the activated eggs were cultured in KSOM at 37°C in 5% CO_2_, and the different stages of the early embryo development process were observed and counted at 6, 24, 48, 72, and 96 hours.

### Microinjection and Measurement of Intracellular Ca^2+^ and Luciferase Expression

Mouse eggs were washed in M2 and microinjected either with complementary RNA (cRNA) or recombinant protein diluted in injection buffer (120 mM KCl, 20 mM HEPES, pH 7.4). All injections were 3% to 5% of the egg volume (10, 12). The cRNA or recombinant protein was mixed with an equal volume of 1 mM Oregon Green BAPTA dextran (Molecular Probes). Eggs were maintained in H-KSOM containing 100 μM luciferin and were imaged on a Nikon TE2000 or Zeiss Axiovert 100 microscope equipped with a cooled intensified CCD camera (Photek Ltd.). Cytoplasmic Ca^2+^ changes were monitored in these eggs for 4 hours after injection by measuring the Oregon Green BAPTA-dextran fluorescence with low-level excitation light from a halogen lamp (11, 14).

At the end of Ca^2+^ measurements, the same set of eggs was then monitored for luminescence (i.e., indicating recombinant protein concentration) by integrating light emission (in the absence of fluorescence excitation) for 20 minutes using the same intensified CCD camera (18, 19). Notably, the fluorescence signals were typically 10 to 100 times greater than the luminescence signals. The Ca^2+^ measurements for an egg were further analyzed only if the same egg was also luminescent. The luminescence reading from eggs was converted into an amount of luciferase by use of a standard curve that was generated by placing eggs in a luminometer that had been previously calibrated by microinjection with known amounts of luciferase protein (Sigma) (8, 21).

### Immunofluorescence of Sperm PLCζ

The anti-PLCζ, V-37, polyclonal antibody was raised in rabbits against a 16-mer-peptide sequence (^8^SKIQDDFRGGKINLEK^23^) of human PLCζ protein and was affinity-purified as per the manufacturer's instructions (Invitrogen). Anti-NusA and anti-β-actin mouse monoclonal antibodies were purchased from Santa Cruz Biotechnology.

Human sperm samples washed with PBS (pH 7.4) were fixed with 4% ethanol-free formaldehyde (Polysciences Inc.) for 30 minutes at 4°C. Fixed samples were resuspended in PBS and spotted onto 0.1% poly L-lysine-coated (Sigma-Aldrich) coverslips and dried for 2 hours at 37°C before permeabilization with 1% Triton X-100 for 1 hour at 23°C. After blocking with 5% normal goat serum (Invitrogen) for 30 minutes, the samples were incubated with V-37 antibody (rabbit IgG in PBS containing 5% normal goat serum) overnight at 4°C, washed with PBS, then incubated with Alexa-488-conjugated goat anti-rabbit antibody (Invitrogen) for 45 minutes. The samples were mounted on slides with antifading reagent (Invitrogen) and observed using a SP5 confocal microscope (Leica) under ×100 oil immersion objective; the collected images were edited with ImageJ (http://rsbweb.nih.gov/ij).

### SDS-PAGE and Immunoblot Analysis

Fresh human sperm samples washed with PBS (pH 7.4) were mixed with 5× SDS sample buffer, vortexed briefly, and sonicated for 5 seconds on ice. Sperm samples and recombinant proteins were separated by SDS-PAGE, as previously described elsewhere (8, 18). Separated proteins were transferred onto polyvinylidene difluoride membrane and incubated overnight at 4°C with the appropriate primary antibody. Detection of horseradish peroxidase-coupled secondary antibody was achieved by use of Super Signal West Dura (Pierce) and a Bio-Rad ChemiDoc gel documentation system for image capture (11, 20).

The human sperm and oocytes used in this study were donated by patients attending the IVF Wales clinic at the University Hospital of Wales, Cardiff, UK. The current project and all associated procedures were approved by the local South East Wales Research Ethics Committee and also by the UK Human Fertilisation and Embryology Authority (R0161).

## Results

### Native and Recombinant Human PLCζ Analysis

**Figure 1:**
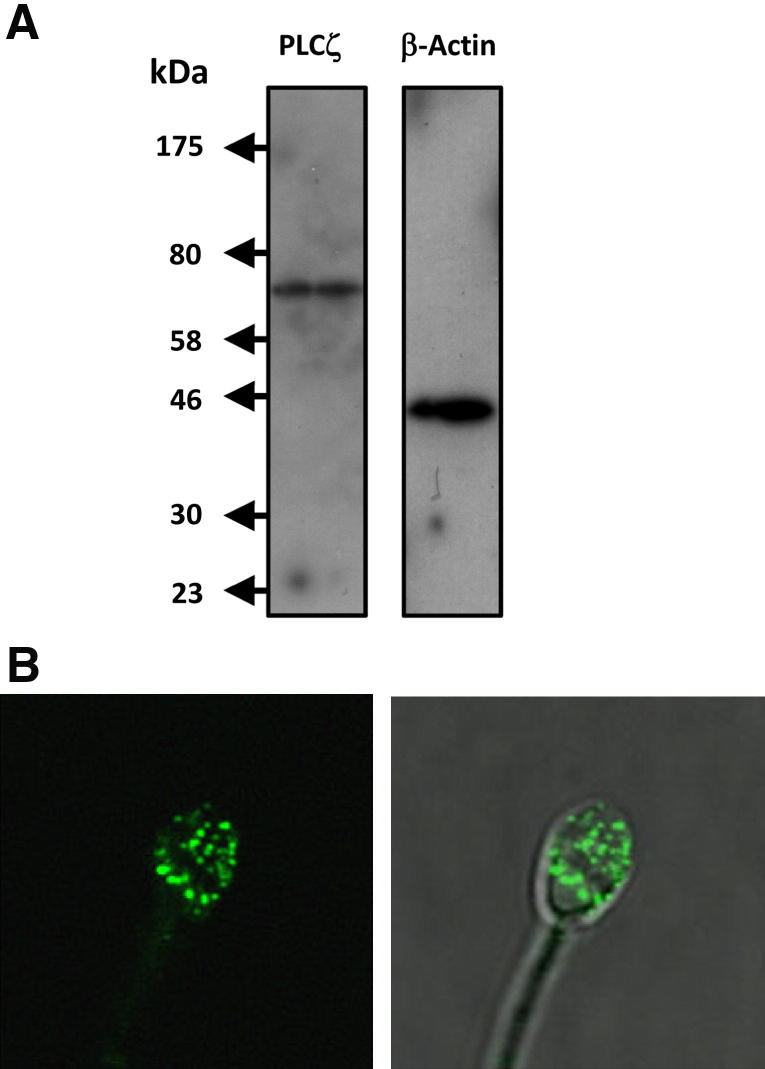
Expression and distribution of phospholipase Cζ (PLCζ) protein in human sperm. (**A**) Immunoblot analysis of PLCζ protein in human sperm. Sperm cells (25,000 per lane) were analyzed by 9% SDS-PAGE, proteins were electrophoretically transferred, and the blot membrane was incubated either with affinity-purified, anti-PLCζ polyclonal antibody (V-37; 1:7,500 dilution; *left panel*) or with anti-β-actin monoclonal antibody (1:2,500 dilution; *right panel*). (**B**) Representative confocal microscope images of PLCζ immunofluorescence in human sperm after fixing and immunostaining with anti-PLCζ polyclonal antibody (V-37; 1:1,000 dilution) showing that the native PLCζ in this IVF patient localizes to the equatorial and acrosomal region of the sperm head.

The expression and distribution of native PLCζ in fertile human sperm was examined by immunoblot and immunofluorescence analysis on ejaculated sperm from a man whose partner had achieved successful pregnancy via ICSI. An affinity-purified, anti-PLCζ polyclonal antibody positively detected a single, immunoreactive 70 kd protein corresponding to human PLCζ (5), with the control anti-β-actin antibody identifying a 42 kd human β-actin band (Fig. 1A). Immunofluorescence analysis revealed native PLCζ localization primarily in the equatorial region of the sperm head with some additional acrosomal staining (see Fig. 1B). Equatorial localization of PLCζ is congruent with fusion of this sperm region with the oocyte plasma membrane at fertilization, thus facilitating early entry of PLCζ into the ooplasm (1–3). The acrosomal staining suggests either an additional role of PLCζ in earlier steps of fertilization that remains undefined, or is due to nonspecific immunoreactivity, although the immunoblot detection of only a single 70 kd protein (see Fig. 1A) would be consistent with the former suggestion.

**Figure 2:**
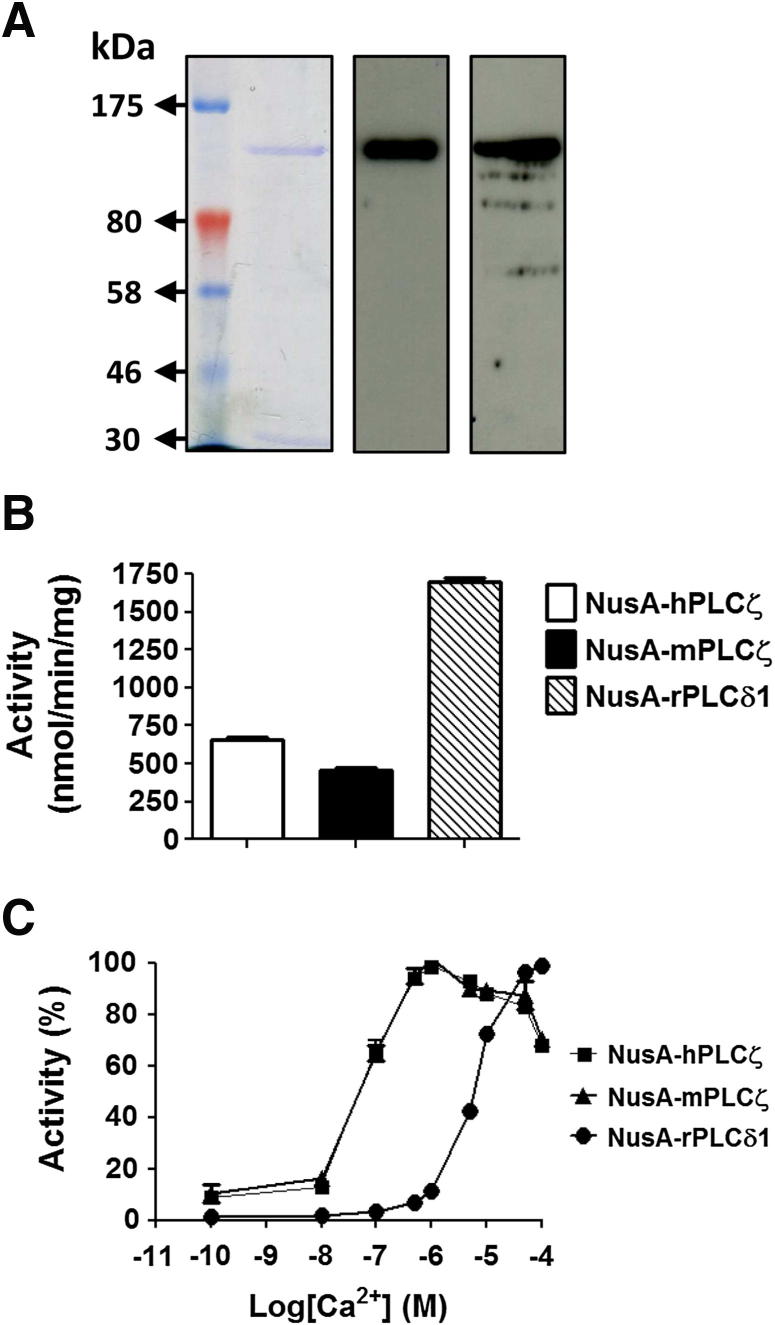
Expression and enzymatic characterization of recombinant wild-type human phospholipase Cζ (PLCζ) protein. (**A**) One μg of bacterially-expressed, affinity-purified NusA-hPLCζ fusion protein analyzed by 7% SDS-PAGE (*left panel*) or by immunoblot analysis with either anti-PLCζ polyclonal (V-37; 1:10,000 dilution; *middle panel*) or anti-NusA monoclonal antibody (1:20,000 dilution; *right panel*). (**B**) The PIP_2_ hydrolysis enzyme activities of recombinant hPLCζ, mPLCζ, and rPLCδ1 purified by nickel affinity chromatography as NusA-fusion proteins (20 pmol) determined with the [^3^H]PIP_2_ cleavage assay, n = 3 ± standard error of the mean (SEM), using two different preparations of recombinant protein and with each experiment performed in duplicate. In control experiments with NusA alone, no specific PIP_2_ hydrolysis activity was observed (data not shown). (**C**) Effect of varying [Ca^2+^] on the normalized PIP_2_ hydrolysis enzyme activity of purified, recombinant hPLCζ, mPLCζ, and rPLCδ1 NusA-fusion proteins. For these assays, n = 2 ± SEM using two different batches of recombinant proteins and with each experiment performed in duplicate.

Recombinant human PLCζ was expressed as a NusA-hexahistidine fusion protein in *E. coli* and purified by Ni-NTA affinity chromatography. Our earlier use of plasmid vectors comprising only the hexahistidine tag (i.e., without a fusion protein, such as NusA) provided reliable recombinant PLCζ protein expression, but it did not effectively yield soluble, functional PLCζ (unpublished data). In contrast, significant expression of soluble NusA-PLCζ was observed, and the affinity-purified human PLCζ fusion protein, after SDS-PAGE and immunoblot analysis, displayed the predicted ∼130 kd molecular mass (NusA ∼60 kd + 70 kd *hPLCζ*) (Fig. 2A). Enzymatic determination of [^3^H]PIP_2_ hydrolysis activity for the purified human PLCζ, mouse PLCζ, and rat PLCδ1 fusion proteins (8, 11, 13, 18) (see Fig. 2B) reveals the human PLCζ to have 42% higher specific activity than mouse PLCζ (655 ± 36 vs. 460 ± 24 nmol/min/mg), but both of these PLCs had much lower specific activity (27% to 38%) relative to *PLCδ1* (1,703 ± 52 nmol/min/mg) (Table 1). The relative Ca^2+^ sensitivity of [^3^H]PIP_2_ hydrolysis was determined between 0.1 nM to 0.1 mM Ca^2+^ (see Fig. 2C), yielding an EC_50_ value for human PLCζ that was near identical with mouse PLCζ (70 vs. 64 nM Ca^2+^), but this was in sharp contrast with the ∼80-fold higher PLCδ1 EC_50_ value of 5,327 nM (see Table 1). The marked EC_50_ disparity is consistent with previous studies of PLC isoform Ca^2+^ sensitivity that indicated that only PLCζ would be near-optimally activated to hydrolyze its PIP_2_ substrate at the ∼100 nM resting Ca^2+^ levels in mammalian eggs (6, 8).

**Figure 3:**
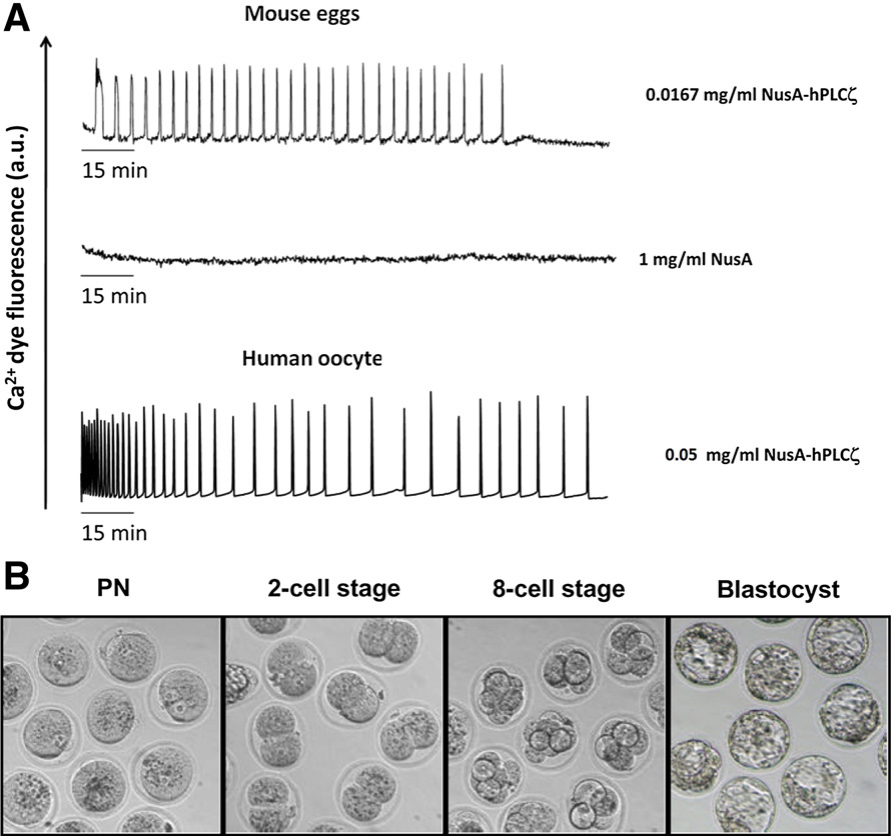
Recombinant human phospholipase Cζ (PLCζ) protein induces Ca^2+^ oscillations in mouse and human eggs and initiates early embryo development. (**A**) Representative fluorescence (au: arbitrary units) recordings reporting the Ca^2+^ concentration changes in a mouse and human egg after microinjection of human PLCζ recombinant protein (*top and bottom trace,* respectively). Microinjection of NusA alone does not induce Ca^2+^ release in mouse eggs (*middle trace*). (**B**) Micrographs illustrating mouse embryos at the various early developmental stages (pronuclear formation [PN], two-cell and eight-cell stages, and blastocyst stage) achieved after egg microinjection with ∼80 fg of purified, wild-type human PLCζ recombinant protein (0.0167 mg/mL). The optimal efficiency of blastocyst formation achieved by microinjection of hPLCζ into mouse eggs was 50% to 60%.

Microinjection of recombinant wild-type human PLCζ into mouse and human eggs revealed that it possesses a potent ability to induce cytoplasmic Ca^2+^ oscillations (Fig. 3A, top and bottom traces, respectively), matching that observed after microinjection of native sperm extracts (2, 3). The NusA protein microinjection alone did not cause any Ca^2+^ changes (see Fig. 3A, middle trace). The minimal PLCζ concentration required for a physiologic pattern of Ca^2+^ oscillations was 0.0167 mg/mL, indicating that the amount of human PLCζ in mouse eggs able to induce Ca^2+^ oscillations and early embryogenesis was ∼80 fg/egg. This is in the same range as the estimated PLCζ content within a single sperm (4). Moreover, we observed that highly efficient early embryo development, from pronuclei formation up to the multicellular blastocyst stage, was also specifically initiated by the human PLCζ protein microinjection (see Fig. 3B). The successful early development to the blastocyst embryo stage observed with wild-type PLCζ-injected eggs was >50% (see Fig. 3B), a value that is very similar to that previously obtained after microinjection of cRNA encoding luciferase-tagged human PLCζ (19).

### In Vivo and In Vitro Analysis of Infertility-Linked PLCζ Mutations

**Figure 4:**
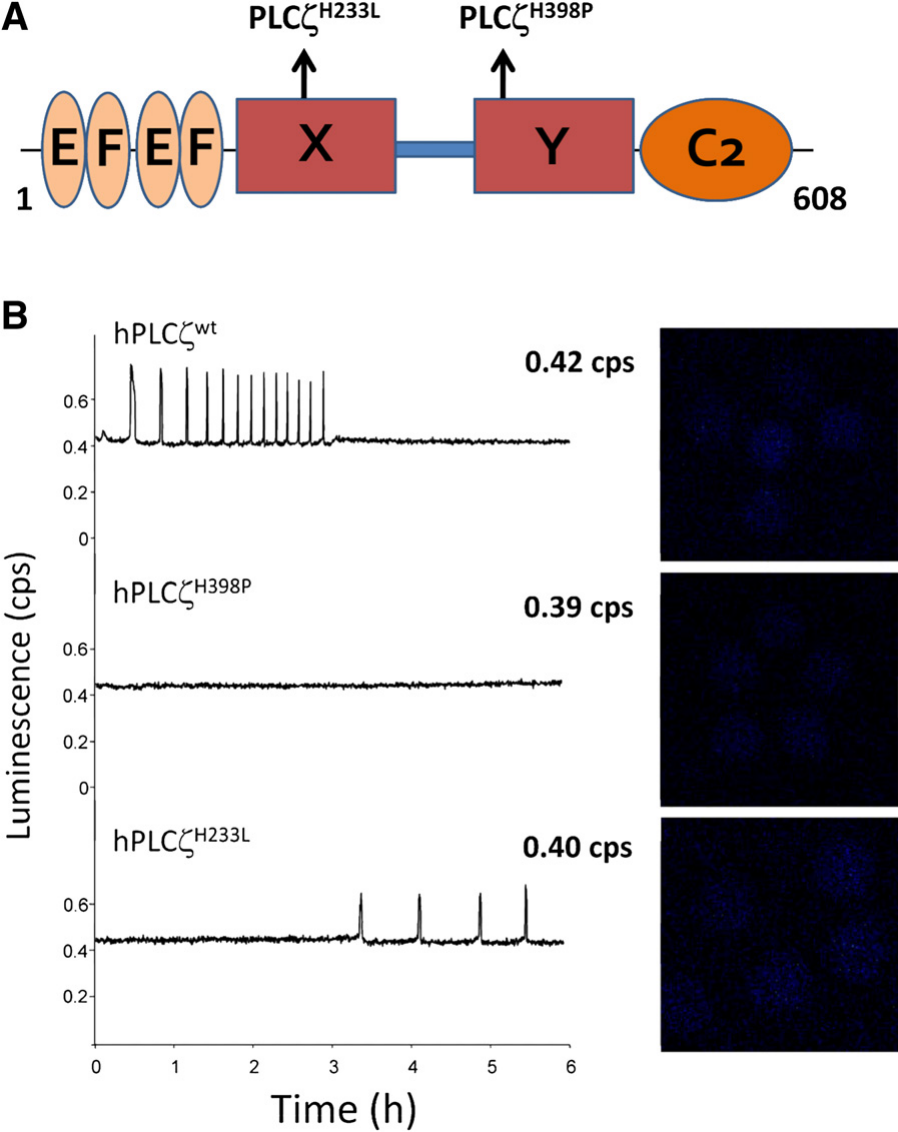
Effect of H233L and H398P mutations on Ca^2+^ oscillation-inducing activity of human phospholipase Cζ (PLCζ) in mouse eggs. (**A**) Schematic representation of human PLCζ domain structure identifying the location of H233L and H398P mutations within the X and Y catalytic domains, respectively. (**B**) Fluorescence and luminescence recordings reporting the cytosolic Ca^2+^ changes (*black traces;* Ca^2+^) and luciferase-PLCζ expression level (in counts per second, cps), respectively, in unfertilized mouse eggs after the microinjection of cRNA encoding luciferase-tagged, wild-type human PLCζ, and the PLCζ^H233L^ and PLCζ^H398P^ mutants. Panels on the right display the integrated luminescence image of individual mouse eggs after cRNA microinjection of either wild-type or mutant PLCζ. The relatively low luminescence values achieved, corresponding to femtogram levels of PLCζ protein expressed in each cRNA-microinjected egg, are intended to mimic the approximate amount of PLCζ that is delivered by entry of a single sperm.

The first direct link between male infertility and a defective PLCζ gene was made after identification of a PLCζ point mutation in an infertile man with failed fertilization after ICSI treatment (16). This PLCζ catalytic domain mutation of a conserved histidine residue to a proline (H398P) (Fig. 4A) disrupts both enzymatic PIP_2_ hydrolysis and Ca^2+^ release activity in mouse eggs (18). A second PLCζ mutation, also in the catalytic domain (H233L) (see Fig. 4A), has recently been identified (17), although this particular histidine residue is not conserved.

To enable the comparison of relative recombinant protein expression by luminescence measurement (8, 21), we prepared luciferase-fusion constructs of each of these human PLCζ mutants as well as wild-type PLCζ for microinjection into mouse eggs. Prominent Ca^2+^ oscillations (∼9 spikes/2 hours) were observed in wild-type PLCζ cRNA-injected mouse eggs, with the first Ca^2+^ spike occurring after ∼25 minutes at a luminescence reading of 0.07 counts per second (Table 2), corresponding to expression of ∼29 fg PLCζ/egg (see Fig. 4B, top trace; see Table 2). Microinjection of mutant PLCζ^H398P^ cRNA totally failed to cause any Ca^2+^ oscillations in mouse eggs (see Fig. 4B, middle trace) (16), consistent with our recent findings for the equivalent mouse mutant (PLCζ^H435P^) (18). It is interesting that, with the other catalytic domain mutation, the PLCζ^H233L^ cRNA produced a dramatic reduction in Ca^2+^ oscillation frequency compared with that of wild type (see Fig. 4B, bottom trace), with only ∼2.8 spikes/2 hours observed (see Table 2). Moreover, there was also a significant delay in initiation of cytoplasmic Ca^2+^ oscillations in the egg, with the first Ca^2+^ spike appearing after ∼190 minutes at a luminescence value of 0.34 counts per second. Hence, whereas PLCζ^H398P^ completely abrogates, the PLCζ^H233L^ mutation substantially reduces the frequency of Ca^2+^ oscillations in mouse eggs, with both resulting in a failure to activate embryo development.

**Figure 5:**
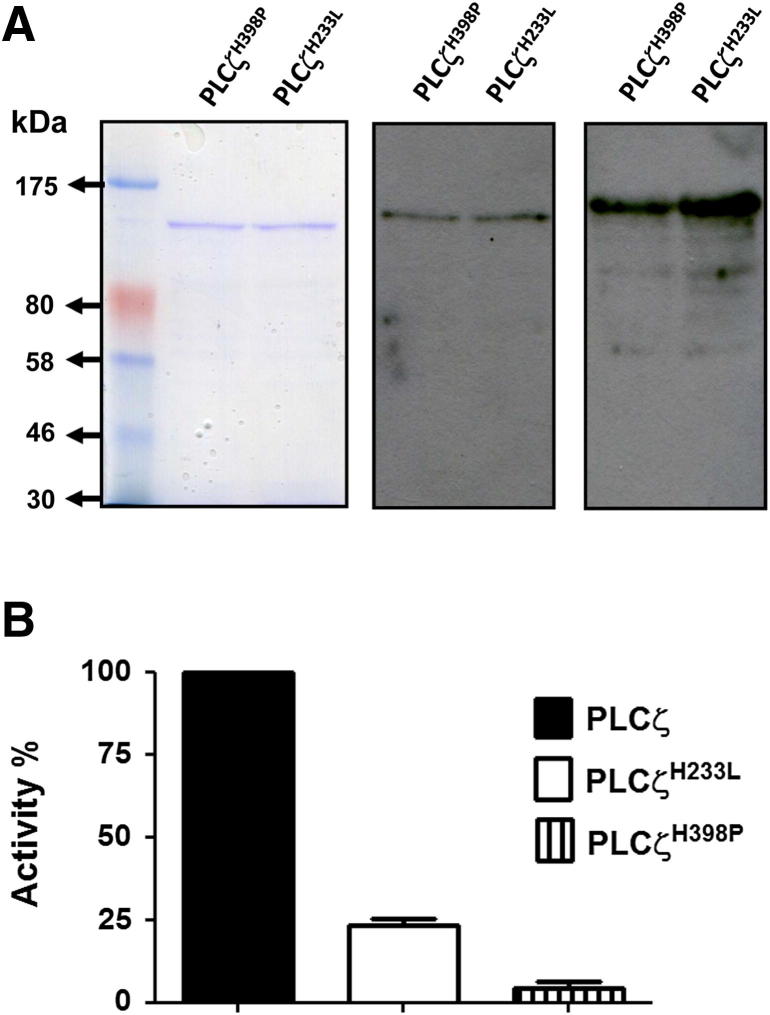
Expression, purification, and enzyme activity of the PLCζ^H233L^ and PLCζ^H398P^ mutant proteins. (**A**) The affinity-purified NusA-fusion proteins for PLCζ^H233L^ and PLCζ^H398P^ (1 μg) analyzed by 7% SDS-PAGE (*left panel*) or by immunoblot analysis using anti-PLCζ polyclonal (V-37; 1:10,000 dilution; *middle panel*) or anti-NusA monoclonal antibody (1:20,000 dilution; *right panel*). (**B**) The [^3^H]PIP_2_ hydrolysis activity of the purified PLCζ^H233L^ and PLCζ^H398P^ proteins, n = 3 ± standard error of the mean, determined using two different preparations of recombinant protein and with each experiment performed in duplicate.

The in vitro PIP_2_ hydrolysis activity of the wild-type human PLCζ, and the PLCζ^H233L^ and PLCζ^H398P^ mutant proteins was compared after their expression in *E. coli,* purification by Ni-NTA affinity chromatography, and gel/immunoblot analysis (Fig. 5A). Enzyme specific activity values obtained for each protein reveal that the PLCζ^H233L^ mutant retains only 24% of the activity of wild-type PLCζ (157 ± 48 vs. 655 ± 36 nmol/min/mg), and the PLCζ^H398P^ mutant almost completely fails to hydrolyze [^3^H]PIP_2_. These enzymatic data indicate that both of these histidine mutations when introduced into human PLCζ dramatically diminish their PIP_2_ hydrolytic activity, thus directly explaining why the cRNA microinjection of these PLCζ mutants into unfertilized mouse eggs fails to induce normal egg activation.

### Rescue of Egg Activation Failure by Microinjection of Human PLCζ Protein

**Figure 6:**
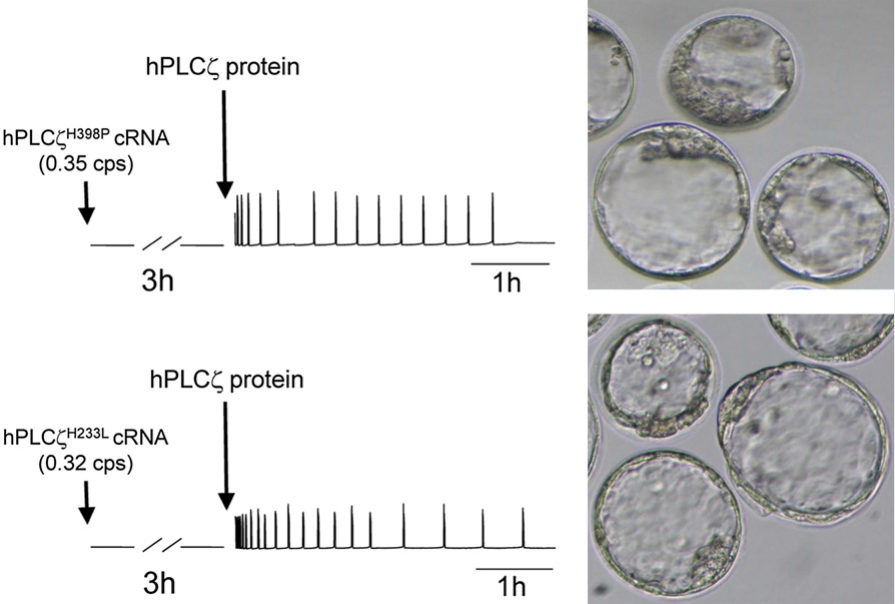
Egg activation failure with mutant forms of human phospholipase Cζ (PLCζ) rescued by microinjection of recombinant, wild-type human PLCζ protein. The traces on the left report the Ca^2+^ concentration changes observed in unfertilized mouse eggs after microinjection with the cRNA for the mutants PLCζ^H398P^ (*short arrow, upper panel*) and PLCζ^H233L^ (*short arrow, lower panel*). After a period of 3 hours to enable femtogram expression of the mutant PLCζ proteins, a second microinjection of ∼80 fg of the affinity-purified, wild-type hPLCζ recombinant protein was performed as described in Figure 3 (*long arrows, upper and lower panels*), approximating the amount of native hPLCζ in a single sperm. The two panels on the right display representative micrographs illustrating the mouse embryos at the blastocyst developmental stage that were observed 96 hours after microinjection of the human PLCζ recombinant protein into each mouse egg.

We further investigated whether the purified, recombinant wild-type human PLCζ protein would be able to rescue the egg activation failure observed after expression of the infertility-linked human PLCζ^H398P^ and PLCζ^H233L^ mutants in mouse eggs (see Fig. 4B, middle and bottom traces, respectively). For this experiment, two different sets of mouse eggs were microinjected with cRNA encoding either the human PLCζ^H398P^ or PLCζ^H233L^ mutant. During the 3-hour time period after the injection of the mutant cRNAs, which enabled both of the mutant PLCζ proteins to be expressed (>0.30 counts per second) at the physiologic level required for fertilization (i.e., the amount of PLCzeta normally present in a single sperm), there were no detectable Ca^2+^ changes observed in either set of mouse eggs (Fig. 6, short arrow-traces on left). At this 3-hour post-cRNA time point, the same eggs were again microinjected, but this time with ∼80 fg of the purified recombinant, human wild-type PLCζ protein.

This intervention with microinjected protein immediately resulted in the highly effective induction of a normal pattern of Ca^2+^ oscillations (see Fig. 6, long arrow-traces in middle), leading to efficient physiologic egg activation and successful early embryo development up to the multicellular blastocyst stage (see Fig. 6, micrographs on right). The efficiency of development to the blastocyst stage for the wild-type PLCζ protein-injected eggs was close to 60%. The observation of efficacious phenotypic rescue of mutant PLCζ-mediated egg activation failure suggests that the direct microinjection of active, wild-type human PLCζ protein could potentially be used as a therapy in specific cases of failed ICSI due to defective PLCζ in human sperm.

## Discussion

Since the discovery of PLCζ a decade ago (4), mounting evidence has strongly supported the notion that sperm-derived PLCζ is the sole physiologic trigger of egg activation during mammalian fertilization (3, 22, 23). Upon sperm-egg fusion, it is believed that PLCζ is introduced into the ooplasm and catalyses PIP_2_ hydrolysis to generate InsP3. The intracellular Ca^2+^ release triggered by InsP3 produces the characteristic cytoplasmic Ca^2+^ oscillations that result in egg activation, and this initiates the embryo development process. Since then, PLCζ has been identified in many different mammalian species, suggesting that it could play a pivotal role at fertilization in all mammals. Furthermore, recent clinical reports have linked reduced protein expression levels and abnormal forms of PLCζ with human male infertility (15–18, 24).

Although ICSI is a powerful technique that is extensively used by IVF clinics to overcome many conditions of male infertility, clinical studies have identified men whose sperm repeatedly fail to fertilize after ICSI due to egg activation failure. The sperm that fail at ICSI cannot induce the Ca^2+^ oscillations required for activation, and recent evidence indicates that this infertile phenotype is associated with defective sperm PLCζ protein in these patients, caused either by a low level of sperm PLCζ protein expression or by genetic mutations resulting in a dysfunctional PLCζ in sperm (15–18, 24).

Despite the major role of PLCζ in mammalian fertilization, thus far only purified recombinant mouse PLCζ has been successfully used to study in vitro biochemical properties and the regulatory mechanisms underlying PLCζ function (6, 8, 9, 11, 13). In this study, we prepared recombinant human PLCζ protein fused to NusA, a fusion protein known to greatly enhance the solubility and stability of recombinant proteins (25). Human PLCζ is present as a 70 kd protein at the equatorial region in sperm (see Fig. 1). The purified human PLCζ protein exhibited higher in vitro PIP_2_ hydrolysis activity than recombinant mouse PLCζ, whereas the EC_50_ for Ca^2+^ sensitivity was very similar for both recombinant proteins (see Fig. 2; see Table 1). Microinjection of recombinant, wild-type human PLCζ protein induced Ca^2+^ oscillations in both mouse and human eggs (see Fig. 3) and successfully activated mouse early embryo development up to the blastocyst stage.

The estimated amount of human PLCζ protein in mouse eggs that was required to efficiently induce Ca^2+^ oscillations and embryogenesis was ∼80 fg/egg (3–5 pL of 0.0167 mg/mL), which is entirely consistent with the PLCζ levels previously shown to be able to trigger egg activation and efficient development of mouse eggs (4, 19). Recombinant mouse PLCζ synthesized by baculovirus expression was less efficient at inducing Ca^2+^ oscillations in mouse eggs compared with recombinant human PLCζ, requiring an estimated 300 fg/egg (6). In our preliminary studies using recombinant human PLCζ expressed alone, without the accompanying presence of a fusion protein to assist in stabilizing enzyme activity, we observed very poor ability to generate Ca^2+^ oscillations. These observations are entirely consistent with the very recent report using human PLCζ expressed without a fusion protein partner that required injection of 5,000 to 10,000 fg/egg to cause Ca^2+^ oscillations and did not result in embryo development to the blastocyst stage (29). Thus, our strategic use of NusA as an efficient fusion protein partner appears to be important for the recovery of significant levels of soluble human PLCζ. Importantly, this enzymatically active PLCζ is capable of effecting successful embryo development when injected into mammalian eggs (see Fig. 3B), via generation of the characteristic Ca^2+^ oscillations that mimic the physiologic egg activation phenomenon observed at fertilization (see Fig. 3A).

To investigate whether injection of recombinant human PLCζ protein would be able to rescue the failed egg activation caused by infertility-linked PLCζ mutants, we assessed the effect of two novel point mutations identified in the PLCζ gene that have previously been specifically linked to male infertility (16, 17, 24). Both of these point mutations, H233L and H398P, are located on the X and Y catalytic domains of human PLCζ, respectively (see Fig. 4A), and they have been found to dramatically reduce in vitro PIP_2_ hydrolysis activity (see Fig. 5B), fully consistent with their inability to produce the normal pattern of Ca^2+^ release in mouse eggs, resulting in egg activation failure (see Fig. 4B). However, microinjection of wild-type human PLCζ protein into mouse eggs that were expressing these infertility-linked PLCζ mutants effectively rescued the failure of egg activation by inducing a normal pattern of Ca^2+^ oscillations, leading to successful early embryo development up to the blastocyst stage (see Fig. 6).

These findings promote the potential application of PLCζ protein into IVF clinics as an effective therapeutic option for egg activation failure due to male factor deficiencies related to PLCζ dysfunction. It has previously been demonstrated that egg activation failure due to defective PLCζ can be approached by using a Ca^2+^ ionophore treatment during ICSI (26), even though this procedure does not specifically induce the characteristic Ca^2+^ oscillations observed at fertilization (27). However, it currently remains to be determined whether such ionophore treatment represents the safest or most effective method for overcoming egg activation failure, as it is known that the precise pattern of Ca^2+^ oscillations after fertilization in mouse eggs can exert potentially deleterious downstream, longer-term effects on both gene expression and embryo development (28).

The co-microinjection of PLCζ cRNA during ICSI could, in principle, be used to rescue egg activation failure of PLCζ-deficient sperm. This method would, however, present difficulties in practice because the rate of synthesis and total amount of PLCζ protein expressed in the egg cannot be readily controlled using a bolus of microinjected cRNA. Previous studies have shown that successful embryo development requires PLCζ to be present within the egg at a relatively precise concentration range to closely match the specific amount of PLCζ that would be provided physiologically by the entry of a single mature sperm at fertilization (19). Thus, the availability of purified, active recombinant human PLCζ protein appears to represent both a highly practical and the most physiologic therapeutic agent for overcoming failed ICSI cases resulting from aberrant sperm PLCζ. Recombinant human PLCζ protein could potentially also be used in regenerative medicine approaches via generation of parthenogenetic embryos and blastocysts that may facilitate stem cell derivation and differentiation.

## Figures and Tables

**Table 1:** Specific enzyme activity and Ca^2+^-dependent [^3^H]PIP_2_ hydrolysis activity of purified NusA-PLC proteins.

NusA-PLC fusion protein	PIP_2_ hydrolytic enzyme activity (nmol/min/mg)	Ca^2+^-dependence of enzyme activity EC_50_ (nM)
Human PLCζ	655 ± 36	70
Mouse PLCζ	460 ± 24	64
Rat PLCδ1	1,703 ± 52	5,327

*Note:* Summary of the specific hydrolytic enzyme activity and the EC_50_ values of Ca^2+^-dependent enzyme activity for [^3^H]PIP_2_ hydrolysis that was determined as described in Materials and Methods; the data were analyzed by nonlinear regression analysis (GraphPad Prism 5) for the affinity-purified NusA-hexahistidine fusion proteins for hPLCζ, mPLCζ, and rPLCδ1 (see Fig. 2B and C).

**Table 2:** Expression of microinjected cRNA encoding luciferase-tagged wild-type phospholipase Cζ (PLCζ) mutants PLCζ^H233L^ and PLCζ^H398P^ in unfertilized mouse eggs.

PLCζ-luciferase injected	Ca^2+^oscillations (spikes/2 h)	Peak luminescence (cps)	Time to 1st spike (min)	Luminescence at 1st spike (cps)
PLCζ^WT^	9.02 ± 0.037	0.42 ± 0.020	∼25	0.07 ± 0.005
PLCζ^H233L^	2.84 ± 0.076	0.40 ± 0.050	∼190	0.34 ± 0.040
PLCζ^H398P^	—	0.39 ± 0.020	—	—

*Note:* Values are mean ± standard error of the mean. The Ca^2+^ oscillation-inducing activity (Ca^2+^ spike number in 2 hours; time to first spike) and the simultaneously-measured PLCζ-luciferase fusion protein luminescence levels (peak luminescence; luminescence at first spike) are summarized for mouse eggs that had been microinjected, as described in Materials and Methods, with cRNA encoding one of the following human PLCζ-luciferase constructs: wild-type PLCζ, the PLCζ^H233L^ or PLCζ^H398P^ mutant (see Fig. 4B). cps = counts per second.
